# Commentary: Identification of Repeat Region Ambiguities in HLA Typing and the Implications for Immunogenetics Research

**DOI:** 10.1111/tan.15768

**Published:** 2024-12-01

**Authors:** Thomas R. Turner, Richard H. L. Natarajan, James Robinson, Steven G. E. Marsh, Neema P. Mayor

**Affiliations:** ^1^ Anthony Nolan Research Institute, Royal Free Hospital London UK; ^2^ UCL Cancer Institute, Royal Free Campus London UK

Next generation sequencing (NGS) has revolutionised HLA typing, offering improvements in coverage, accuracy, resolution and throughput. Illumina and Ion Torrent platforms provide many short, but highly accurate sequence reads, while PacBio and Oxford Nanopore Technologies platforms generate fewer but much longer sequence reads. The lower raw accuracy of long reads is mitigated by consensus sequence generation and crucially allows phasing of polymorphisms across the entire lengths of the HLA genes. Combinations of short and long read sequencing technologies are also in use, utilising the advantages of both techniques to further improve accuracy. Together, NGS technologies are driving rapid increases in the numbers of alleles, many as full‐length genomic sequences, submitted to the IPD‐IMGT/HLA Database. Submissions from hitherto less well characterised HLA genes has also grown, enriching the database as a whole.

Sequencing technologies, and the bioinformatic tools used alongside them, have advantages and disadvantages [1] but they all, to differing extents, struggle to sequence through repetitive regions accurately and consistently. Within the HLA genes, these repeat regions are largely, but not exclusively, confined to introns and in their simplest form can be just three or more of the same consecutive nucleotide (homopolymers). The longest homopolymer currently recorded in a gene on the IPD‐IMGT/HLA Database (v3.57, 07/2024) is a 45 bp long stretch of Thymine nucleotides in MICB. In HLA, the longest is a 34mer of Thymines in HLA‐DRB1*15:01:01:26. Generally, the longer the homopolymer, the lower the probability of determining the correct numbers of bases, with underestimation (deletions) more common than overestimation (insertions) due to signal merging. Other repeats involve longer nucleotide motifs where the error mode is more complex (microsatellites). These predominantly occur in the expansive introns of the HLA class II genes and help contribute to their large and variable lengths, particularly for the HLA‐DRB genes.

During full‐length HLA sequencing of the International HLA and Immunogenetics Workshop (IHIW) cell lines [2] and UK haematopoietic cell transplant (HCT) donors and recipients [3, 4], we uncovered widespread novel intronic polymorphism. As per the requirements for submission of novel alleles to the IPD‐IMGT/HLA Database, we repeated the PCR and sequencing to confirm novel nucleotide positions. In some cases, different intronic variants of the same coding allele were observed. On closer inspection, these variants differ only subtly by their repeat region lengths. To further identify these alleles, alignments were performed for intronic variants of each coding sequence on the IPD‐IMGT/HLA Database. An allele was eliminated from the list if we observed unique SNPs, or indels not in a repeat region. We interpreted the remaining clusters of alleles as repeat region ambiguities (RRAs) of each other, replacing the initial typing with a short RRA string (e.g., DPB1*04:01:01:01/02/04/06/14/35). While crucial to confidently assigning typing to our samples, this task was laborious, somewhat subjective and potentially error prone. The challenge of accurately processing and reporting any mismatches across RRA regions is handled in a number of ways by the multiple bioinformatics tools available, with varying degrees of accuracy [5].

The existence of repetitive regions in the HLA genes represents natural genetic variation. There are, however, limitations in currently available technologies to determine numbers of repeats accurately and consistently. Signal merging and enzyme slippage during PCR amplification or sequencing, as well as overlapping of short sequence reads, can all contribute to this issue. Adding to the complexity of RRAs is that they are subject to the nuances of each individual workflow, which varies between laboratories. Combinations of different PCR setups, sequencing chemistries and analysis software will potentially generate different sets of RRAs, which will be influenced by the molecular landscape surrounding the repeat regions. Additionally, certain pairs of alleles may be RRAs of another but not each other. For example, three different allele sequences may have 7x, 8x or 9x repeats of a motif. Any of these allele sequences could be represented as an exact match to itself, or to either of the other two alleles, with appropriate indels. Importantly, all three of these alleles could be real and any two combinations of them could exist in a heterozygous individual. Another challenge is the dynamic interpretation of repeat regions indels compared with existing reference sequences. This is something that could vary with each database release as more alleles are added. By interpreting groups of alleles as RRAs, we are not doubting they are real, but are taking a pragmatic, if conservative approach, to qualify limitations in technology which will be experienced by many.

Increasingly, NGS based HLA typing assays are being used to submit novel alleles to the IPD‐IMGT/HLA Database, and as such RRA numbers will continue to grow with every database release. On average, 1.55% of alleles with full‐length genomic sequences are RRAs, but this varies between genes. Currently, 0.08% of HLA‐B alleles would be considered RRAs, rising to 6.7% in HLA‐DRB1. It would be contentious and impractical to restrict submissions of alleles that only differ by repeats, as they could be real and meet submission requirements. It would however be prudent for submitters to be aware of this and err on the side of caution when preparing to submit these alleles, perhaps even performing additional checks. Most full‐length genomic sequences in the IPD‐IMGT/HLA Database have been derived from non‐IHIW cells, largely through bulk submissions by donor registries. The identification of RRAs within a relatively small cell line panel emphasises the importance of IHIW cells for establishing robust and consistent interpretation of HLA typing data across the field. Indeed, the 17th IHIW cell line quality control component acknowledged these ‘concordant ambiguities’ [6].

There has been a recent drive towards routine use of NGS‐HLA typing, with mounting evidence that matching at higher resolution has clinical impact [7, 8, 9, 10, 11]. However, while full‐gene coverage is now commonplace in HLA typing workflows, there is often hesitance to assign 4‐field HLA types for some loci, largely due to the complexity of repeat regions. While repeat region lengths have well defined clinical impact in some diseases, including Huntingdon's disease and Fragile X syndrome [12], the implications of false interpretation are broader in immunogenetics. For example, if individuals are assigned different alleles within RRA strings, the degree of matching between patients and donors will be reduced, or the allele frequencies in a population will be skewed. The H&I community is making great strides to establish NGS‐HLA typing as a routine clinical diagnostic tool, for the many advantages that are familiar to us. However, there will increasingly be consequences for H&I laboratories as well as population genetic and clinical studies if RRAs are ignored. So, as we strive to improve our understanding of HLA and other hyperpolymorphic, immune‐related genes, often with patients in mind, we should not let the complexities of repeat regions undo our progress. We hope that this article sparks a broad and inclusive discussion of how to identify and interpret RRAs, leading to a consensus on their impact on our fields of study and a way to mitigate their negative impact going forward.

## 1 Conflicts of Interest

The authors declare no conflicts of interest.
